# Dotinurad versus benzbromarone in Japanese hyperuricemic patient with or without gout: a randomized, double-blind, parallel-group, phase 3 study

**DOI:** 10.1007/s10157-020-01849-0

**Published:** 2020-01-24

**Authors:** Tatsuo Hosoya, Takafumi Sano, Tomomitsu Sasaki, Masahiko Fushimi, Tetsuo Ohashi

**Affiliations:** 1 Jikei University School of Medicine, 3-25-8, Nishi-Shimbashi, Minato-ku, Tokyo, 105-8461 Japan; 2 Medical R&D Division, Development Department, Fuji Yakuhin Co., Ltd., 4-383, Sakuragi-cho, Omiya-ku, Saitama-shi, Saitama, 330-9508 Japan

**Keywords:** Hyperuricemia, Uricosuric, Selective urate reabsorption inhibitor, Dotinurad, FYU-981, Benzbromarone

## Abstract

**Background:**

Dotinurad is a novel selective urate reabsorption inhibitor that reduces serum urate levels in hyperuricemic patients with or without gout by selectively inhibiting urate transporter 1. This study was conducted to compare the efficacy and safety of dotinurad with those of benzbromarone.

**Methods:**

In this 14-week, randomized, multicenter, double-blind, parallel-group, dose escalation, benzbromarone-controlled, phase 3 study, hyperuricemic patients with or without gout were randomized to two groups that received either dotinurad 2 mg or benzbromarone 50 mg. Dotinurad or benzbromarone was administered once a day for 14 weeks. The primary endpoint was the percent change in serum uric acid level from the baseline to the final visit.

**Results:**

A total of 201 Japanese hyperuricemic patients with or without gout (dotinurad: 102, benzbromarone: 99) received at least one dose of the study drug. The mean percent change in serum uric acid level from the baseline to the final visit in the dotinurad and benzbromarone groups was 45.9% and 43.8%, respectively. Non-inferiority of dotinurad 2 mg to benzbromarone 50 mg in lowering serum uric acid was verified by the predefined non-inferiority margin (95% CI − 1.27 to 5.37%). The incidence of adverse events and adverse drug reactions was comparable between the two groups.

**Conclusion:**

Dotinurad 2 mg was verified to have a non-inferior serum uric acid lowering effect compared with benzbromarone 50 mg, in Japanese hyperuricemic patients with or without gout.

**ClinicalTrials.gov Identifier:**

NCT03100318.

## Introduction

Japanese guidelines for the management of hyperuricemia and gout define hyperuricemia as a serum uric acid level > 7.0 mg/dL [1]. Hyperuricemia is a causative factor for urate deposition diseases such as urolithiasis and gouty arthritis. Additionally, recent studies have shown that hyperuricemia has a close relationship to lifestyle diseases such as chronic kidney disease, hypertension, and diabetes mellitus [2–4]. In the Japanese guidelines, medication is recommended for hyperuricemia without gout or gouty tophi (asymptomatic hyperuricemia), complicating these lifestyle diseases in cases where the serum uric acid level is ≥ 8.0 mg/dL. For gouty and gouty tophi patients, the aim of treatment is to maintain serum uric acid levels ≤ 6.0 mg/dL thereby dissolving urate crystals in the joints [1].

The serum uric acid level is maintained by balancing production and excretion. Hyperuricemia is thus classified mainly into “overproduction type”, “underexcretion type” or “combined type”, according to uric acid clearance and amount of urinary uric acid excretion [5]. The proportion of each type is 10%, 60%, and 30% respectively, in Japan. However, the recently revised guideline (third edition) classifies hyperuricemia into three types: “underexcretion type”, “renal load type”, or “combined type” [1]. The renal load type, which has recently been proposed, is further divided into two subtypes: “overproduction type” and “extrarenal underexcretion type”. It has become evident that the extrarenal underexcretion type is likely to increase uric acid excretion from the kidneys [6], resulting in apparent overproduction [1]. Therefore, it can be assumed that overproduction due to a metabolic disorder occurs in less than 10%, and most hyperuricemic patients in Japan have impaired uric acid excretion.

Japanese management guidelines recommend use of xanthine oxidase inhibitors (XOI), such as allopurinol, febuxostat and topiroxostat for “overproduction type” and uricosuric drugs, such as probenecid and benzbromarone, for “underexcretion type” [1]. Although many Japanese hyperuricemic patients have characteristic of underexcretion, uricosuric drugs have been not often used to treat such patients [7]. Benzbromarone, which was the most frequently used uricosuric drug in Japan, is available in a few countries, and is prohibited in patients with hepatic impairment because serious hepatic impairment, including fulminant hepatitis, was observed [8]. Lesinurad, a selective urate reabsorption inhibitor (SURI), which is URAT1 inhibitor without affecting other urate transporters such as OAT1 and OAT3 has recently been approved in the United States and the European countries [9]. It is indicated in combination with an XOI for gouty patients who failed to achieve a target serum uric acid level because serious acute kidney injury was observed with high-dose lesinurad monotherapy in a clinical study [10]. Furthermore, the serum uric acid lowering effects of some uricosuric drugs are weakened in patients with the complication of moderate to severe renal impairment [1]. For these reasons, hyperuricemic patients with renal impairment have mainly used XOIs. As described above, uricosuric drugs have some safety concerns; the development of safer drugs with sufficient serum uric acid lowering effect is therefore expected [7].

Dotinurad is a novel SURI for treatment of hyperuricemia with or without gout. The concept behind the development of dotinurad is to improve on the safety problems seen with benzbromarone, such as hepatic impairment, while maintaining a strong serum uric acid lowering effect. Previous studies showed that dotinurad is efficacious and safe [NCT02344862, NCT02416167]. We thus considered it necessary to compare dotinurad with benzbromarone. This study was conducted to compare the efficacy and safety of dotinurad 2 mg/day with those of benzbromarone 50 mg/day and to verify non-inferiority of its serum uric acid lowering effect in Japanese patients with hyperuricemia, with or without gout.

## Methods

### Study design

This phase 3, 14-week, randomized, multicenter, double-blind, parallel-group, dose escalation, benzbromarone-controlled, comparative study was conducted at 17 clinical institutions in Japan.

### Inclusion and exclusion criteria

The inclusion criteria were a serum uric acid level during the run-in period ≥ 7.0 mg/dL (patients with a history of gouty arthritis or gouty tophus), ≥ 8.0 mg/dL (patients with asymptomatic hyperuricemia who had a diagnosis or were treated for hypertension, diabetes, and/or metabolic syndrome), or ≥ 9.0 mg/dL (asymptomatic hyperuricemic patients without the aforementioned complications). All were Japanese outpatients aged 20 years or older on the day of written informed consent obtained for participation in this study. The serum uric acid level criteria were followed by the Japanese management guidelines [5].

The exclusion criteria were as follows: gouty arthritis that had not became asymptomatic within the 2 weeks before the day of randomization; possible disorders causing secondary hyperuricemia (e.g., Lesch-Nyhan syndrome, psoriasis vulgaris, hypothyroidism); hemoglobin A1c (HbA1c; NGSP) ≥ 8.4%; use of drugs that might have affected the outcome of this study during the 2 weeks before the starting day of the run-in period to randomization; hyperuricemia classified as “overproduction type” or an indeterminate; complications of any serious cardiac disorder; a history of myocardial infarction, and/or an angina attack within a year; complications or a history of cancer (in the 5 years before obtaining informed consent); complications of hepatic impairment or aspartate aminotransferase (AST) and/or alanine aminotransferase (ALT) ≥ 100 U/L; complications of renal calculus or clinical manifestations suspicious of a urinary calculus (e.g., hematuria, back pain); estimated glomerular filtration rate (eGFR) < 30 mL/min/1.73 m^2^; blood pressure ≥ 180 mmHg systolic and/or ≥ 110 mmHg diastolic; complications of stroke or stroke within a year before obtaining informed consent; a history of drug allergy including allergy to benzbromarone; and presence of any other clinically significant medical conditions that could potentially preclude participation in this study. If patients had been treated with any antihyperuricemic agent or drugs affecting the serum uric acid level before the enrolment of this study, they were allowed to enter into this study only after a washout period of 2–4 weeks.

### Treatment

**Fig. 1:**
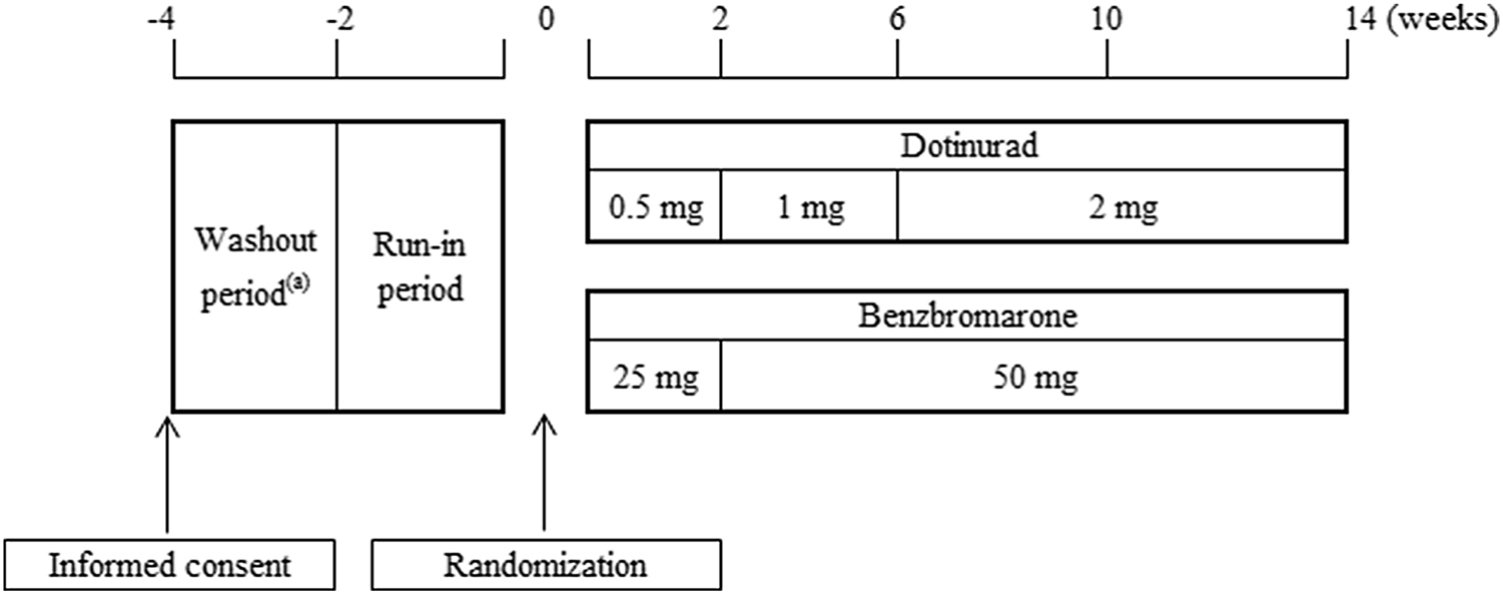
Dosing schedule. ^(a)^Patients who had been treated with uric acid lowering drugs or treatment affecting the serum uric acid level were subjected to the wash-out period

Figure 1 shows the dosing protocol. At the end of the run-in period, patients were randomly assigned to dotinurad or benzbromarone (1:1 ratio). An independent organization conducted randomized block allocation of the study drug. To minimize the risk of gouty arthritis due to rapid serum uric acid reduction, the dose titration method was adopted [11]. Most hyperuricemic patients treated with benzbromarone have a stably controlled serum uric acid level at a dose of 25–50 mg/day, thus the initial and maintenance doses of benzbromarone were set to 25 mg/day and 50 mg/day, respectively [12]. The initial dose of benzbromarone was administered for the first 2 weeks, and the maintenance dose was administered from weeks 2–14. The initial dose of dotinurad was 0.5 mg/day for the first 2 weeks and then 1 mg/day for 4 weeks. As a maintenance dose, 2 mg/day of dotinurad was administered from weeks 6–14.

Furthermore, to minimize the risk of a urinary calculus in association with increased urinary uric acid excretion, a urinary alkalization drug (e.g., citrate) was given together with the study drug in the following cases: (1) history of urolithiasis, (2) urine pH < 6.0 (from obtaining of informed consent to the end of this study), and (3) needs for the therapy at an investigator’s discretion.

To maintain double-blind conditions, the serum uric acid level was not disclosed to the patients, study investigators, and local sponsor personnel from the study drug administration until the final database was disclosed.

### Classification of hyperuricemia

In this study, the classification of hyperuricemia was implemented according to the second edition of the Japanese management guideline [5], the latest version when the study started. Hyperuricemia was classified into four types based on measurement of uric acid in a 60-min urine collection during the run-in period: (1) uric acid overproduction type—urinary extraction of uric acid (*E*_UA_) > 0.51 mg/kg/h and uric acid clearance (*C*_UA_) ≥ 7.3 mL/min/1.73 m^2^; (2) uric acid underexcretion type—*E*_UA_ < 0.48 or *C*_UA_ < 7.3; (3) combined type—*E*_UA_ > 0.51 and *C*_UA_ < 7.3; and (4) normal type—0.48 ≤ *E*_UA_ ≤ 0.51 and *C*_UA_ ≥ 7.3. The patients classified as “uric acid overproduction type” were excluded from this study, for fear of the urinary calculus formation.

### Efficacy endpoints

The primary efficacy endpoint was the percent change in serum uric acid level from the baseline to the final visit. The secondary efficacy endpoints were the percentage of patients achieving a serum uric acid level ≤ 6.0 mg/dL at the final visit and the serum uric acid levels at each time point.

### Safety evaluations

Adverse events (AEs) and safety assessments were conducted by clinical investigators based on vital signs, 12-lead electrocardiography, clinical laboratory tests, and clinical examinations throughout this study. AEs were classified according to the system organ class and preferred term (MedDRA version 20.1; Japanese Maintenance Organization, Tokyo, Japan) and were evaluated in terms of their potential causal relationship with the study drug, as well as severity and seriousness. AEs judged to be related to the study drug were defined as adverse drug reactions (ADRs).

### Statistical analyses

We assumed that the percent changes in serum uric acid level with dotinurad 2 mg and benzbromarone 50 mg were 42% and 37% [12], respectively. When we determined the number of patients required to detect the non-inferiority of dotinurad 2 mg to benzbromarone 50 mg based on these percent changes in serum uric acid levels with a standard deviation (SD) of 10% in each group, a simulation with one-sided significance set at 2.5% and detection power of 90% indicated that each group would require 10 patients. Taking into consideration the number of patients who might be excluded from the analyses and those in whom safety could be evaluated, we set the group size to 100 patients in each group.

All analyses of efficacy were evaluated using the full analysis set (FAS), which comprised all randomized patients who received at least one dose of the study drug and underwent serum uric acid measurement during at least one visit. If the serum uric acid level at the last visit (week 14) was missing, this data would be compensated for by the last observation carried forward (LOCF) method. This approach was prespecified before the start of this study. In the efficacy analyses of the primary endpoint, the mean values between the dotinurad and the benzbromarone groups were compared using the two-sample *t* test. The non-inferiority margin was calculated as 10%, which was less than one-third of the difference between benzbromarone 50 mg and placebo in terms of the serum uric acid lowering effect. Assessment of the non-inferiority of dotinurad 2 mg to benzbromarone 50 mg was based on a prespecified non-inferiority margin. In the analyses of the secondary efficacy endpoints, Fisher’s exact test and two-sample t-tests were used to compare mean values between each group.

Safety analyses were evaluated for the safety population (SP), which comprised all patients who took at least one dose of the study drug. The incidence of AEs was summarized as the number and proportion of patients. Fisher’s exact test was used to compare incidences between each group.

SAS software, version 9.2 (SAS Institute, Cary, NC, USA) was used to perform all statistical analyses of efficacy and safety. Unless otherwise specified, all values are expressed as mean ± SD. The statistical significance was defined based on a two-tailed *P* value of < 0.05.

## Results

### Patient flowcharts and baseline characteristics

**Fig. 2:**
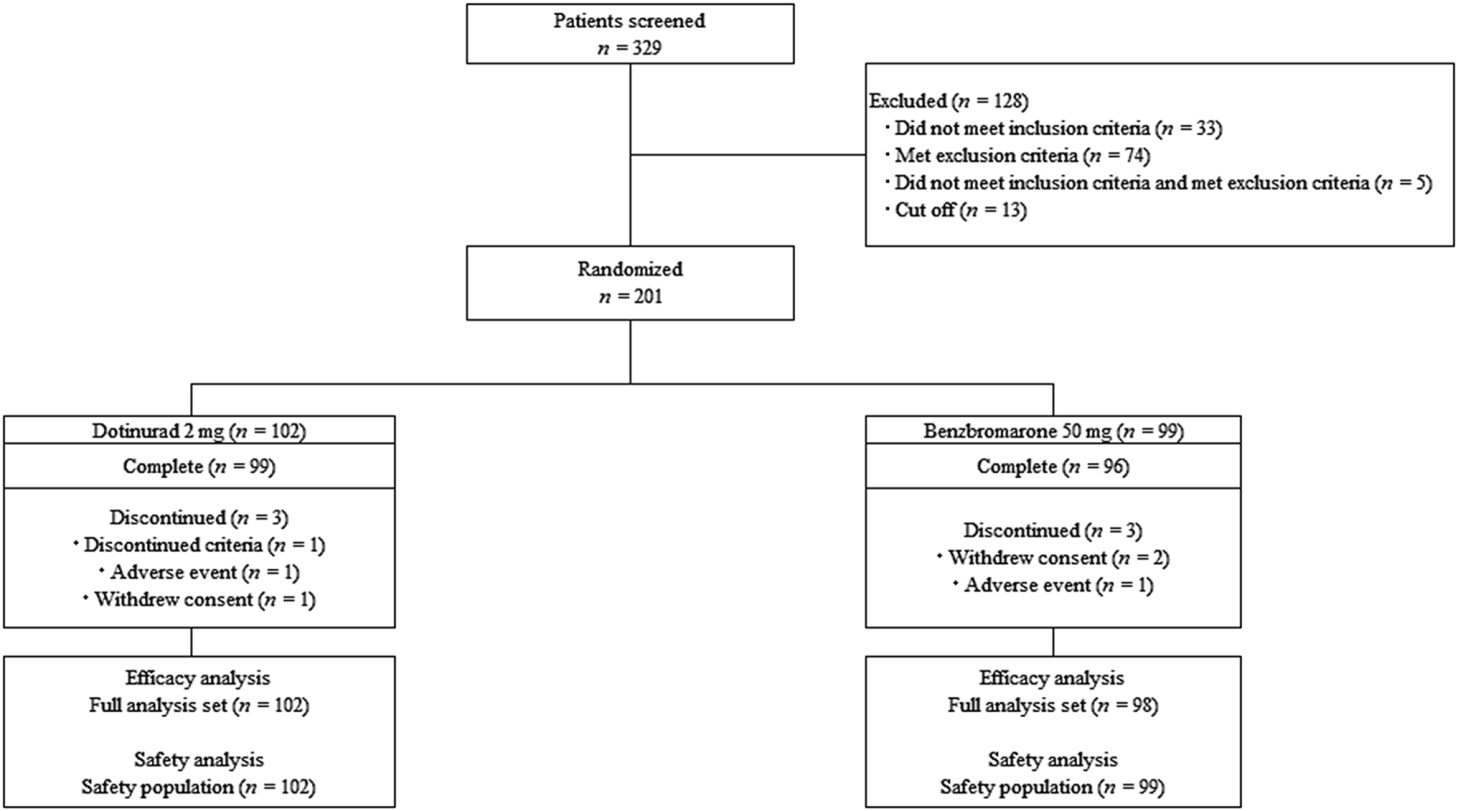
Flow diagram of study protocol

Figure 2 summarizes the flow diagram of study protocol. Within the period of April 2017 to August 2018, 329 patients were screened, 128 were excluded, and the remaining 201 were randomized to receive the dotinurad (*n* = 102) or the benzbromarone (*n* = 99). Six patients discontinued the study. One patient in benzbromarone group was not adopted from the efficacy analyses because the patient met the exclusion criteria after the administration. All patients who received at least one dose of the allocated drug were included in the SP.

The baseline characteristics of patients were comparable between the two groups. Mean serum uric acid level during the run-in period was 8.90 and 8.92 mg/dL in the dotinurad and benzbromarone groups, respectively. Mean eGFR was 65.3 and 66.2 mL/min/1.73 m^2^, respectively (Table 1).

**Table 1:** Baseline characteristics of patients enrolled

Characteristic		Dotinurad	Benzbromarone
		(*n* = 102)	(*n* = 98)
Sex	Male	100 (98.0)	98 (100.0)
	Female	2 (2.0)	0 (0.0)
Age (year)	Mean ± SD	55.0 ± 10.3	54.7 ± 10.4
Height (cm)	Mean ± SD	170.9 ± 6.7	168.9 ± 7.1
Weight (kg)	Mean ± SD	75.7 ± 13.3	74.8 ± 12.6
Serum uric acid (mg/dL)	Mean ± SD	8.90 ± 1.16	8.92 ± 1.28
eGFR (mL/min/1.73 m^2^)	Mean ± SD	65.3 ± 11.5	66.2 ± 12.1
Medical history of hyperuricemia	Number of patients (%)	74 (72.5)	75 (76.5)
History of gouty arthritis	Number of patients (%)	83 (81.4)	77 (78.6)
Existence of gouty tophus	Number of patients (%)	1 (1.0)	0 (0.0)
Drinking habits	Number of patients (%)	67 (65.7)	73 (74.5)
Classification of hyperuricemia	Uric acid underexcretion type (%)	91 (89.2)	90 (91.8)
	Combined type or normal type (%)	11 (10.8)	8 (8.2)

eGFR for male (mL/min/1.73 m^2^) = 194 × Serum creatinine^−1.094^ × Age^−0.287^

eGFR for female (mL/min/1.73 m^2^) = 194 × Serum creatinine^−1.094^ × Age^−0.287^ × 0.739 [17]

Definition of drinking habit: consumption of alcohol more than 3 days of the week and consumption of more than 500 mL beer or 60 mL of whisky in a day

### Efficacy

#### The primary efficacy endpoint

The percent changes (mean ± SD) in serum uric acid level from the baseline to the final visit were 45.9 ± 11.9% and 43.8 ± 11.8% in the dotinurad and benzbromarone groups, respectively (Table 2). The difference in percent change between the two groups was 2.0% (95% CI − 1.27 to 5.37). Because the lower limit of the 95% CI of the between-group difference was higher than the prespecified non-inferiority margin, non-inferiority of the serum uric acid lowering effect of dotinurad 2 mg in comparison to benzbromarone 50 mg was confirmed. In addition, a significant difference in percent change in serum uric acid levels from the baseline to the final visit was not observed in two groups (*P *= 0.224; two-sample *t* test).

**Table 2:** Result of primary and secondary efficacy endpoints

Endpoint	Category	Dotinurad (*n* = 102)	Benzbromarone (*n* = 98)
Percent change in serum uric acid level from the baseline to the final visit	Mean ± SD (%)	45.9 ± 11.9	43.8 ± 11.8
	95% Confidence Interval	43.57 to 48.27	41.49 to 46.24
	95% Confidence Interval of difference between groups	− 1.27 to 5.37	
	Two-sample *t* test	*P *= 0.224	
Percentage of patients with serum uric acid level ≤ 6.0 mg/dL at the final visit	Number (%)	88 (86.2)	82 (83.6)
	95% Confidence Interval	78.04 to 92.29	74.84 to 90.37
	Fisher’s exact test	*P *= 0.693	

Two-sample *t* test and Fisher’s exact test were adjusted with respect to dotinurad group versus benzbromarone group

#### The secondary efficacy endpoint

The percentages of patients achieving a serum uric acid levels ≤ 6.0 mg/dL at the final visit were 86.2% (88/102 patients) and 83.6% (82/98 patients) in the dotinurad and benzbromarone groups, respectively (Table 2), and significant differences were not observed (*P *= 0.693; Fisher’s exact test).

**Fig. 3:**
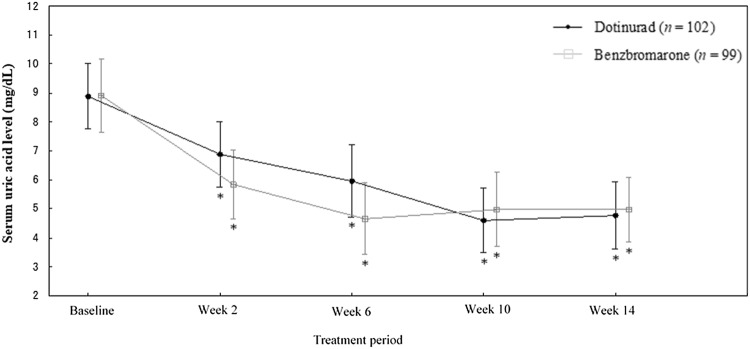
Changes in serum uric acid level in response to follow treatment with dotinurad and benzbromarone. Error bars indicates standard deviation

Figure 3 shows the changes in serum uric acid level in response to follow treatment with dotinurad and benzbromarone. Mean serum uric acid level at the final visit in the dotinurad and benzbromarone groups were 4.80 and 4.98 mg/dL, respectively. At week 2 and 6, the serum uric acid lowering effect of benzbromarone was significantly greater than that of dotinurad (*P* < 0.001; two-sample *t* test). In contrast, the effect of dotinurad was significantly greater than that of benzbromarone at week 10 (*P* = 0.022; two-sample *t* test).

#### Safety

Table 3 shows the incidences of AEs and ADRs in this study. The incidence of AEs was 45.1% in the dotinurad group and 40.4% in the benzbromarone group (Table 3). No significant difference was found between the dotinurad and benzbromarone groups (*P *= 0.569; Fisher’s exact test). Most AEs were mild or moderate in severity. Gastric cancer, judged to be a serious AE, was observed in one case in the benzbromarone group. The investigator ruled out a causal relationship between the study drug and this event because the gastric cancer discovered after the initiation of study drug administration was in its terminal phase and therefore must have begun to develop before the start of this study.

**Table 3:** Incidences of adverse events and adverse drug reactions

Group	Dotinurad (*n* = 102)				Benzbromarone (*n* = 99)				Fisher’s exact test
	Events	Patients	Incidence (%)	95% CI	Events	Patients	Incidence (%)	95% CI	*P* value
AEs	68	46	45.1	35.2–55.3	66	40	40.4	30.7–50.7	*P *= 0.569
ADRs	17	15	14.7	8.5–23.1	19	15	15.2	8.7–23.8	*P *= 1.000
Gouty arthritis	8	8	7.8	3.4–14.9	5	5	5.1	1.7–11.4	*P *= 0.569

Fisher’s exact test was conducted in the dotinurad 2 mg group and benzbromarone 50 mg group

Incidence (%) = number of patients/number of analyzed patients × 100

95% CI: 95% confidence interval

AEs for which patients discontinued the study, with the exception of serious AEs, were observed in two patients (gouty arthritis and bursitis) in the dotinurad group and in one patient (blood creatinine increased, blood urea increased, haemoglobin decreased, red blood cell decreased, and haematocrit decreased) in the benzbromarone group. These AEs in the dotinurad group were judged by the investigator to be ADRs, while a causal relationship between these AEs and benzbromarone was ruled out.

The incidence of ADRs in the dotinurad and benzbromarone groups was comparable, and no significant difference was observed (*P *= 1.000, Fisher’s exact test) (Table 3).

The incidence of gouty arthritis was 7.8% in the dotinurad group, and 5.1% in the benzbromarone group (Table 3). No significant difference was observed between the two groups (*P *= 0.569, Fisher’s exact test). The severity of gouty arthritis was mild or moderate, and all were judged as ADRs by the investigator.

## Discussion

Non-inferiority of the serum uric acid lowering effect of dotinurad 2 mg in comparison to benzbromarone 50 mg was confirmed by the percent change in serum uric acid level from the baseline to the final visit. There was no significant difference in the percentages of patients achieving a serum uric acid level ≤ 6.0 mg/dL at the final visit between both groups.

In the kidney, uric acid is filtered through the glomeruli and then excreted with both reabsorption and secretion. URAT1 is a urate transporter involved in reabsorption, and ABCG2, OAT1, and OAT3 are transporters involved in urate secretion. Furthermore, ABCG2 is involved in the excretion of urate from the gastrointestinal tract. Previous reports reveal that benzbromarone inhibits not only URAT1 but also ABCG2, OAT1, and OAT3 [13–15]. On the other hand, in our non-clinical study, we confirmed that dotinurad inhibited URAT1 selectively, and the inhibition of other urate transporters, namely, ABCG2, OAT1 and OAT3, were very weak. Furthermore, we showed that the IC50 of URAT1 inhibition in dotinurad and benzbromarone were 0.0372 mM and 0.190 mM, respectively in this study. Although the effect of URAT1 inhibition in dotinurad was approximately 5 times more potent than that of benzbromarone in vitro study, the uric acid lowering effect of dotinurad at 1 mg/kg was approximately equal to that of 30 mg/kg benzbromarone in Cebus monkeys [15]. From the above, we anticipate that dotinurad can result in more efficient uric acid excretion without inhibiting excretion from the gastrointestinal tract and uric acid secretion in the kidney. Therefore, we consider that efficient uric acid excretion is one of the factors why dotinurad has a serum uric acid lowering effect at a lower dose than benzbromarone.

Regarding safety, no significant difference was found in the incidence of AEs between the dotinurad and benzbromarone groups. Serious AEs were not observed in the dotinurad group, and although one case of gastric cancer defined as a serious AE occurred in the benzbromarone group, a causal relationship between benzbromarone and the event was ruled out.

Benzbromarone has been reported to cause hepatic impairment as an ADR [8]. In Japan, therefore, even in patients without hepatic impairment, regular liver function testing is recommended during the first six months after the start of benzbromarone administration. In this study, the incidence of ADRs related to hepatic impairment (AST increased and ALT increased) was 2.0% (four events in two patients) in the benzbromarone group. However, the mean values of AST, ALT, and γ-GTP did not significantly change after benzbromarone administration. On the other hand, no ADRs related to hepatic impairment and significant change of hepatic parameters were observed in the dotinurad group (Table 4). In a clinical pharmacology study of dotinurad for subjects with hepatic impairment, major pharmacokinetic and pharmacodynamic parameters and safety were almost the same as those in normal subjects, except *C*_max_ in the group with severe hepatic impairment [NCT03306667]. Furthermore, although the nature of the hepatic impairment observed with benzbromarone suggests that it is not caused by its URAT1 inhibiting effect, but may be originated from its chemical structure [16], dotinurad was structurally designed to avoid the hepatotoxicity observed with benzbromarone. These factors support this contention that dotinurad is less likely to cause hepatic impairment.

**Table 4:** Summary of laboratory data

			Dotinurad (*n* = 102)		Benzbromarone (*n* = 99)	
	Standard range	Visit	Mean ± SD	95% CI	Mean ± SD	95% CI
AST (U/L)	10–40	Run-in period	25.1 ± 7.3	23.7–26.6	24.6 ± 8.2	22.9–26.2
		Final visit	25.6 ± 9.4	23.7–27.4	24.4 ± 9.1	22.6–26.2
ALT (U/L)	5–45	Run-in period	26.5 ± 11.4	24.3–28.7	26.3 ± 14.5	23.4–29.2
		Final visit	27.3 ± 15.1	24.3–30.2	25.1 ± 15.0	22.1–28.1
γ-GTP (U/L)	< 79	Run-in period	52.4 ± 36.2	45.3–59.5	59.3 ± 45.7	50.2–68.5
		Final visit	52.6 ± 38.2	45.1–60.1	52.4 ± 42.9	43.8–60.9
Serum creatinine (mg/dL)	0.65–1.09	Run-in period	0.963 ± 0.147	0.934–0.992	0.961 ± 0.164	0.928–0.994
		Final visit	0.953 ± 0.167	0.920–0.986	0.956 ± 0.183	0.920–0.993
eGFR^a^	–	Run-in period	65.3 ± 11.5	63.07–67.61	66.2 ± 12.0	63.76–68.57
(mL/min/1.73 m^2^)		Final visit	66.4 ± 12.8	63.97–69.00	66.8 ± 13.0	64.25–69.47

^a^eGFR at the final visit was calculated by Post hoc analysis

95% CI: 95% confidence interval

Some uricosuric drugs are known to be less effective in patients with renal impairment, and lesinurad has been reported to cause severe renal impairment. As a result of subgroup analysis of renal function (normal: eGFR ≥ 90 mL/min/1.73 m^2^, mild: eGFR ≥ 60 to < 90 mL/min/1.73 m^2^, moderate: eGFR ≥ 30 to < 60 mL/min/1.73 m^2^), the serum uric acid lowering effect of dotinurad was barely affected by renal dysfunction, even that of moderate degree (Table 5). With respect to AEs related to renal impairment in this study, blood creatinine increased was observed in 2.0% (two events in two patients) in the dotinurad group and 1.0% (one event in one patient) in the benzbromarone group. These events were mild or moderate, and no causal relationship with the study drugs was found. Therefore, dotinurad has been suggested to be a beneficial agent in hyperuricemic patients with moderate renal impairment.

**Table 5:** Serum uric acid lowering effects by eGFR at the baseline

eGFR^a^ category	Dotinurad			Benzbromarone		
	*n*	Percent change ± SD^b^ (95% CI)	Achievement rate^c^ (95% CI)	*n*	Percent change ± SD^b^ (95% CI)	Achievement rate^c^ (95% CI)
Moderate	32	46.8 ± 11.9 (42.56–51.16)	84.38 (27/32) (67.21–94.72)	30	41.6 ± 12.7 (36.94–46.43)	83.33 (25/30) (65.28–94.36)
Mild	69	45.9 ± 11.5 (43.14–48.69)	88.41 (61/69) (78.43–94.86)	67	44.9 ± 11.4 (42.12–47.72)	83.58 (56/67) (72.52–91.51)
Normal	1	16.0	0 (0/1) (0.00–97.50)	1	38.8	100.00 (1/1) (2.50–100.00)

^a^eGFR category, normal: eGFR ≥ 90 mL/min/1.73 m^2^, mild: eGFR ≥ 60 to < 90 mL/min/1.73 m^2^, moderate: eGFR ≥ 30 to < 60 mL/min/1.73 m^2^

^b^Percent change in serum uric acid level from the baseline to the final visit

^c^Percentage of patients with serum uric acid level ≤ 6.0 mg/dL at the final visit

SD: standard deviation; 95% CI: 95% confidence interval

In conclusion, dotinurad 2 mg/day was verified to have a non-inferior serum uric acid lowering effect compared with benzbromarone 50 mg/day. We anticipate that dotinurad can be given to hyperuricemic patients, with or without gout, as a novel antihyperuricemic agent with adequate efficacy and fewer safety concerns.
